# Computed Tomography Prevalence of Cervicothoracic Endplate Junction Alterations in Dogs

**DOI:** 10.3390/ani15081171

**Published:** 2025-04-18

**Authors:** Carles Planas Padrós, Anna R. Tellegen, Henk van den Broek, Stefanie Veraa

**Affiliations:** Division of Diagnostic Imaging, Department of Clinical Sciences, Faculty of Veterinary Medicine, 3584 CL Utrecht, The Netherlands

**Keywords:** endplate, annulus fibrosus, osteochondrosis, avulsion

## Abstract

Spinal degenerative changes can predispose back pain and neurological deficits in dogs. In humans and veterinary patients, the endplate junction has been proposed as an alternative site of failure in addition to damage of the annulus fibrosus, leading to intervertebral disc herniation. Some alterations lead to contour abnormalities with or without regional mineralizations and can be classified as Endplate Junction Failure (EPJF), osteochondrosis (with or without fragmentation), or spondylarthritis. The objective of this retrospective study was to describe the prevalence of endplate junction alterations (EPJA) on CT in dogs at the cervicothoracic junction and classify alterations based on recent presumed EPJF grading. Computed tomographic scans of 315 dogs that included the cervicothoracic (C6–T2) spine for a variety of reasons obtained between January 2020 and December 2022 were assessed for the presence of alterations, location and type. Other spinal abnormalities were also described. A total of 945 intervertebral disc spaces were evaluated, and EPJA were found in 11 intervertebral disc spaces (1.16%). C6–C7 and C7–T1 intervertebral disc spaces were equally affected (five cases each), with only one case affecting T1–T2. In conclusion, cervicothoracic endplate alterations were anecdotically observed on CT in a population of dogs.

## Simple Summary

The intervertebral disc acts as a cushion for the spine and as a flexible transition between the non-flexible vertebral bodies. The intervertebral disc attaches to the vertebral body at the endplates, and alterations in the endplate junction could predispose to spinal degeneration and intervertebral disc weakness, potentially leading to the presence of pain and/or herniation. Computed tomographic studies of the cervicothoracic spine of dogs were reassessed to evaluate the presence of endplate junction alterations that could potentially lead to intervertebral disc degeneration. Abnormalities in the annular–vertebral junction were only visualized in 1.16% of evaluated intervertebral disc spaces.

## 1. Introduction

The intervertebral disc (IVD) is constituted of two parts: the nucleus pulposus (NP) and the annulus fibrosus (AF). The AF surrounds the NP, consists of concentric fibrous rings of collagen, and is thicker at its ventral aspect. An endplate is present between the IVD and the vertebral bodies, with the presence of two endplates (cranial and caudal) for each IVD. The endplate consists of an osseous and hyaline cartilage layer and acts as a transition zone between the disc and the adjacent vertebra [1,2]. Due to its location, endplates play an important biomechanical role. Also, since only the outer layers of the AF are supplied by blood vessels, the endplates play an important nutritional role, with oxygen and glucose diffusing from the vertebrae to the NP across the endplate [1]. Because of its location, endplates are predisposed to mechanical failure. Minor damage to the endplate can lead to progressive structural changes to the adjacent IVD, with a secondary increased risk of disc damage and protrusion or extrusion. Together with endplate changes, other regional anatomical abnormalities, such as changes to the IVD, vertebral bone or soft tissues, can lead to discogenic pain and/or disc herniation, with possible spinal cord compression [3,4]. Intervertebral disc (IVD) degeneration is a common finding in aging dogs, its prevalence and location varying between chondrodystrophic and non-chondrodystrophic breeds. Within the cervical spine, the location for herniation depends on the dog’s breed type. While chondrodystrophic dogs such as Dachshunds and Beagles are predisposed to cranial cervical (C2–3) herniations, dogs from large non-chondrodystrophic breeds more often suffer from caudal cervical disc herniations [5,6]. Since most CSM abnormalities are centred on C4–C7, the presence of caudal cervical herniations is also related to the presence of cervical spondylomyelopathy (CSM) [7,8,9].

Similar to the lumbosacral junction, the cervicothoracic acts as the transition between the more flexible cervical spine and the relatively rigid thoracic spine, comparable to the flexible lumbar spine and fused sacral vertebrae. The cervicothoracic junction, therefore, is an interesting area for biomechanics of the spine. The caudal cervical spine supports most of the torsion movements of the cervical region, leading to continuous insults and tension to the caudal cervical endplates, which can develop into regional IVD damage and subsequent herniation [10,11,12]. The anatomical transition is at C7–T1; however, the functional transition extends cranially and caudally to the adjacent discs.

Both in human and veterinary medicine, the assumption has been that disc herniation occurs through AF failure (AFF), either by direct dorsal protrusion of the AF or by a defect in the AF leading to the extrusion of NP material. However, more recent publications [4,13] propose that endplate junction failure (EPJF) can be a leading cause of disc herniation, evidenced by a larger number of human patients with lumbar disc herniation caused by EPJF compared to AFF in one study. Initial publications [14] were focused on human medicine, with more recent veterinary publications describing similar abnormalities at the lumbosacral junction in dogs [13,15,16].

The aim of the current study was to evaluate the prevalence of endplate junction alterations (EPJA) in a population of dogs that underwent CT examination for a variety of reasons and grade the alterations based on a grading system created for presumed EPJF. The hypothesis is that the presence of EPJA in the cervicothoracic junction will be visualized in a subset of the included dogs and that it will be related to the presence of intervertebral disc degeneration and other spinal abnormalities.

## 2. Materials and Methods

### 2.1. Study Design and Case Selection

This study was a retrospective cross-sectional study. The Picture Archiving and Communication System (PACS; Impax, Version 8.1.2 SP3 HF5, Agfa N.V., Mortsel, Belgium) of the Division of Diagnostic Imaging, Department of Clinical Sciences, Faculty of Veterinary Medicine, Utrecht University, The Netherlands, was used to search Computed Tomographic (CT) studies from January 2020 until December 2022 that included the cervicothoracic spine. The hospital information software (Enterprise Imaging 8.2.2.050, Agfa Healthcare, Rijswijk, The Netherlands; Provet 2.11.5, Espoo, Finland) was used to retrieve data from the medical records, including breed, body weight and age at the time of CT imaging, sex, and castration status. For statistical analysis, dogs were grouped into four body weight classes (small, <10 kg; medium, 10–24 kg; large, 25–49 kg; giant, >50 kg) and three age classes (adolescent, 1–2 years old; adult, 3–7 years old; senior, >7 years old). Dog breeds were also classified based on being chondrodystrophic or non-chondrodystrophic, following the same classification system as in the paper by Tellegen et al. [13].

Inclusion criteria were availability of the cervical spine from C6 to T2 in the CT examination (but not per se area of interest), including bone setting with slice thickness ≤1 mm and soft tissue setting, and availability of patient history and signalment. In dogs with multiple CT examinations, only the most recent one was included. Exclusion criteria were defined as previous surgery in the cervicothoracic region, presence of concurrent spinal conditions that could confound the diagnosis (neoplasia and vertebral fractures), <1 year of age at the time of the examination, scans not completely including the C6–T2 region, and scans with no available bone setting or slice thickness in bone setting >1 mm. The patient selection process was made by a second-year resident of the European College of Veterinary Diagnostic Imaging (ECVDI) (C.P.) and agreed upon by two board-certified ECVDI diplomates (S.V., A.T.).

Images from client-owned dogs were used with the consent given and anonymity preserved, in accordance with hospital policy with reference to research. Hence, approval by the Institutional Animal Care and Use Committee was not required.

### 2.2. Scanning Protocol

Computed Tomography scans were performed with a 64-slice sliding gantry CT scanner (Siemens Somatom Definition AS, Siemens Healthcare, The Hague, The Netherlands) with the dogs under general anaesthesia. Positioning varied according to the region of interest and dog status (dorsal or sternal recumbency), with preserved spinal alignment avoiding excessive extension or flexion. Scanning parameters (including kVp, mAs, tube rotation time, spiral pitch, and matrix) and field-of-view varied according to dog size and region of interest for the scan.

In all dogs, reconstructions of 0.6 mm or 1 mm thick slices for bone (B50f) reconstruction kernel and 2 mm for soft tissue (B30f) reconstruction kernel were made in a transverse plane, while some patients also had available sagittal reconstructions with the same slice thickness and kernels as the transverse. When needed, multiplanar reconstructions (MPR) were made to align the endplates with the image planes (dorsal and/or sagittal). Images were reviewed in soft tissue (window width 300, window level 50) and bone (window width 3000, window level 600) settings.

### 2.3. Data Collection

The CT scans were reviewed by a second-year ECVDI resident (C.P.). The hospital information system and PACS were assessed to determine whether cases met the inclusion criteria based on signalment (age, breed, body weight, and sex) and clinical history. The clinical diagnosis and other reasons for undergoing the CT scan were also recorded.

Subsequently, CT scans that met the inclusion criteria were evaluated for endplate alterations in a blinded manner, with signalment and clinical history concealed. If endplate junction alterations (EPJA) were suspected by the initial reviewer, the findings were confirmed by consensus with two board-certified veterinary radiologists (S.V., A.T.).

**Figure 1:**
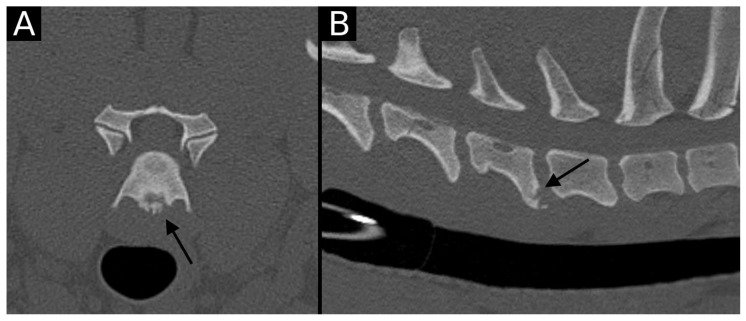
Transverse (**A**) and sagittal (**B**) reconstructions in bone algorithm. An irregular contour surrounded by mild sclerosis (arrow) is observed at the ventral aspect of the C6 caudal endplate, consistent with EPJA Type A. Mild spondylosis deformans is observed in the same vertebra.

**Figure 2:**
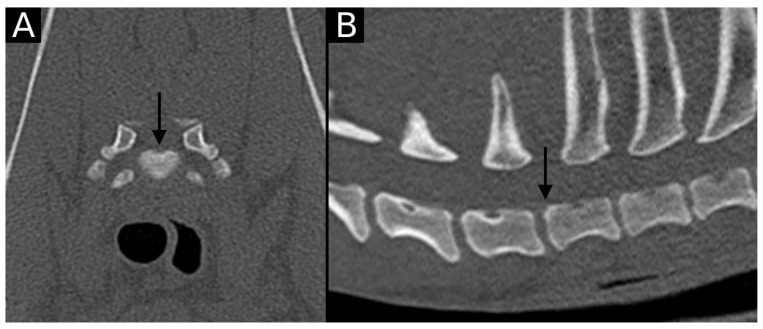
Transverse (**A**) and sagittal (**B**) reconstructions in bone algorithm. Mild contour irregularity and flattening lined by mild sclerosis are observed at the dorsal aspect of the T1 cranial endplate (arrow), consistent with EPJA Type A.

**Figure 3:**
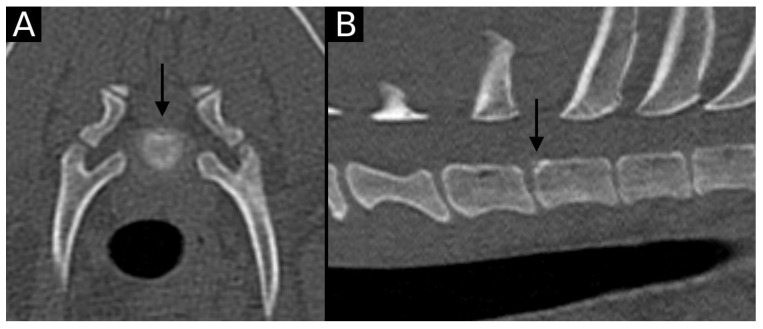
Transverse (**A**) and sagittal (**B**) reconstructions in bone algorithm. Contour irregularities lined by mild sclerosis are observed at the dorsal aspect of the T1 cranial endplate. A small, non-displaced mineral structure is observed at the same level (arrow). This is consistent with EPJA Type B.

**Figure 4:**
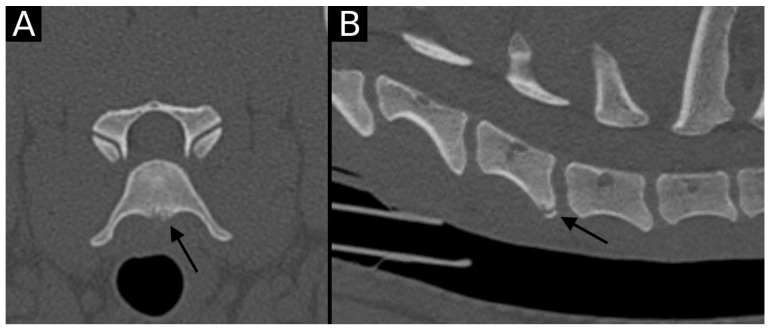
Transverse (**A**) and sagittal (**B**) reconstructions in bone algorithm. The ventral aspect of the caudal endplate of C6 has a well-defined, mildly irregular contour defect lined by mild sclerosis. A well-defined bone fragment (arrow) is observed mildly ventrally displaced with respect to the endplate. This is compatible with EPJA Type C.

The junction between the endplates and intervertebral discs of the cervicothoracic junction (C6–C7, C7–T1 and T1–T2) were evaluated for the presence of alterations. When present, they were also graded and scored for EPJA, IVD herniation, spondylosis deformans, endplate sclerosis, articular process joint osteoarthrosis, and the presence of vertebral morphological abnormalities. The scoring system for endplate junction alterations and other spinal abnormalities was based on the proposed scoring by Tellegen et al. [13] for presumed EPJF at the lumbosacral spine. Endplate junction alterations were classified into four types (A, B, C, D): Type A involved presence of irregularity of the endplate margins without an obvious free fragment (Figure 1 and Figure 2); Type B involved avulsion evidenced by a thin rim of bone associated with a defect in the dorsal edge of the adjacent endplate (Figure 3); Type C included frank avulsion of a bony fragment (Figure 4) and Type D included presence of bony avulsion of both endplates. If EPJA was present, location (dorsal or ventral) and lateralization of the defect were also recorded. IVD herniation was graded from grades 0 to 3: grade 0 indicated no to mild (0–24%) herniation; grade 1 indicated mild to moderate (25–49%) herniation; grade 2 indicated moderate to marked (50–74%) herniation; grade 3 indicated marked herniation to complete obliteration (75–100%) of the vertebral canal by disc material. Spondylosis deformans was scored from grades 0 to 3: grade 0 indicated no enthesophytes; grade 1 indicated a small enthesophyte at the edge of the epiphysis that does not extend past the endplate; grade 2 indicated the presence of an enthesophyte that extends beyond the endplate but does not connect to the adjacent vertebra; and grade 3 indicated connecting enthesophytes from adjacent vertebrae forming a mineralized bridge. The other mentioned abnormalities (IVD mineralization, endplate sclerosis and articular process joint osteoarthrosis) were scored as present (1) or absent (0).

### 2.4. Statistical Analysis

Statistical analysis was performed using commercially available software (IBM SPSS Statistics, version 29.0, IBM Corp., Armonk, NY, USA). Continuous clinical data is presented with the median. Descriptive statistics reported the prevalence, type, and location of presumed EPJF. Next to the presence of EPJA, the following variables were included in the analyses: age, sex (including neutered status), body weight, and presence of concurrent vertebral abnormalities (endplate sclerosis, IVD mineralization, IVD herniation, articular process joint osteoarthrosis, and presence of morphological abnormalities). The distribution of numerical variables was assessed for normality by Kolmogorov–Smirnov or Shapiro–Wilk tests. To test the independence, groups were compared using either the Student’s *t*-test (2 groups) or ANOVA analysis (>2 groups) with the previous application of a Levene test. For the non-parametric tests, the Mann–Whitney U test (2 groups) or Kruskall–Wallis tests (>2 groups) were used. When assessing independence between two non-parametric tests, the Chi-square test was used. A chi-square test of presumed EPJA with each of the determinants was also applied, with a 95% confidence interval (CI).

## 3. Results

### 3.1. Included Cases

Following inclusion criteria, a total of 315 dogs were included in the study. The population consisted of 149 females (from which 88 were neutered) and 166 males (from which 73 were neutered). Age ranged from 1 to 20 years of age (median 5.8 years), distributed in 112 adolescent dogs, 70 adult dogs, and 133 senior dogs. Weight ranged from 1.8 to 87 kg (median 28.3 kg), distributed in 31 small, 84 medium, 196 large and 4 giant dogs. Of the included dogs, 225 were purebred (71.4%), and 90 (28.6%) were crossbreed dogs. The most represented breeds (more than five dogs per breed) were as follows: Labrador Retriever (n = 62), Golden Retriever (n = 19), Belgian Shepherd (n = 10), German Shepherd (n = 9), American Staffordshire (n = 8), Rottweiler (n = 8), King Poodle (n = 7), Labradoodle (n = 6), Dachshund (n = 6), and Rhodesian Ridgeback (n = 5). A total of 55 other breeds were included in the study, with groups ranging from one to four dogs (Supplementary Table S1). Of the included purebred dogs, 25 were from chondrodystrophic breeds, and 200 were from non-chondrodystrophic breeds.

### 3.2. Location and Grading of EPJA

**Figure 5:**
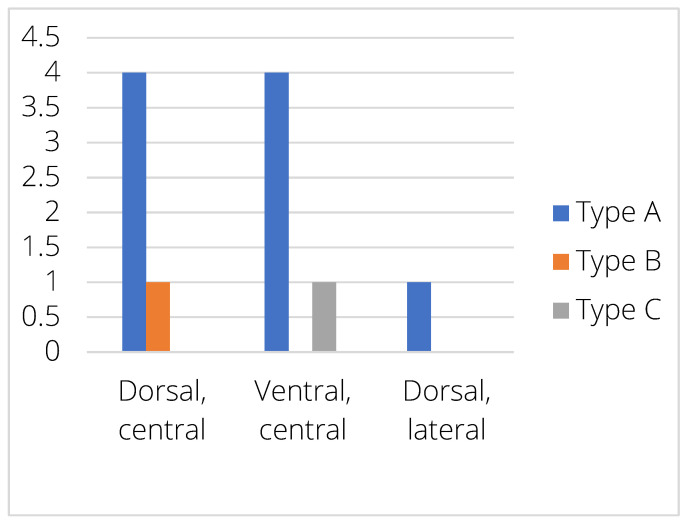
Distribution of the different EPJA types within the endplate.

A total of 11 intervertebral disc spaces of 10 different dogs presented EPJA along the cervicothoracic junction, with the following grading distribution: Type A—nine cases; Type B—one case; and Type C—one case. No Type D were observed in the present study. The location of the lesion was ventral–central in five cases (four Type A, one Type C), dorsal–central in five cases (four Type A, one Type B), and dorsal–lateral in one case (Type A) (Figure 5).

**Figure 6:**
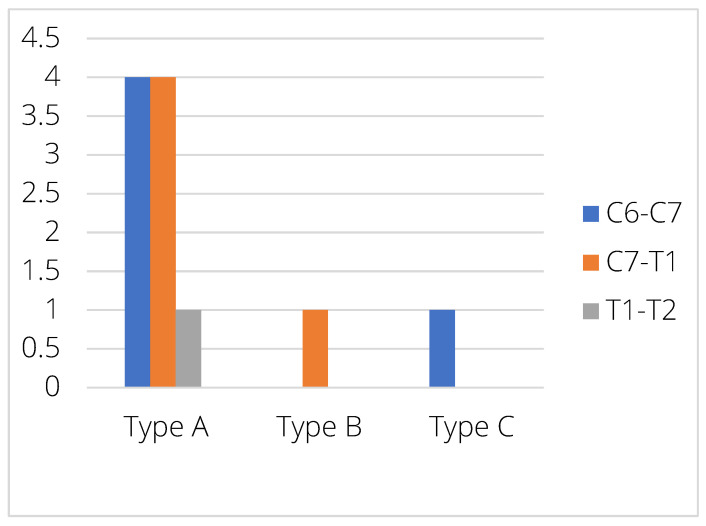
Distribution of the different EPJA types between the different intervertebral disc spaces.

When distributed along the different IVD levels, five EPJA were observed in C6–C7, five at C7–T1, and one at T1–T2. At C6–C7, four cases were Type A and one case was Type C. At C7–T1, four cases were Type A and one case Type B. At T1–T2, the only case was Type A (Figure 6).

### 3.3. Other CT Abnormalities

**Figure 7:**
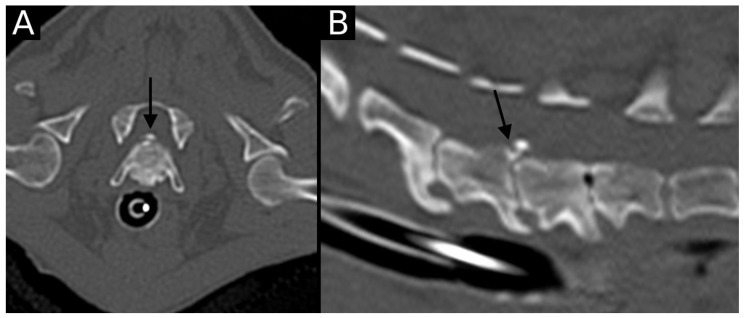
Transverse (**A**) and sagittal (**B**) reconstructions in the bone algorithm in the only dog with an intervertebral disc herniation. The C6–C7 intervertebral space is markedly narrowed, with the presence of moderate to marked spondylosis deformans, mild endplate sclerosis and mineralized herniated disc material. An irregularly defined contour defect is observed at the dorsal aspect of the caudal endplate of C6 (arrow). This was classified as EPJA Type A. The adjacent intervertebral disc spaces are also narrowed, with endplate sclerosis and spondylosis deformans. The vacuum phenomenon is also observed at C7–T1.

Endplate sclerosis was observed in 38 dogs, distributed in 13 dogs at C6–7, 19 at C7–T1 and 6 at T1–T2. The vacuum phenomenon was only observed in seven dogs, distributed in four at C6–C7, two at C7–T1, and one at T1–T2. A similar distribution was observed in facet joint osteoarthrosis: a total of eight dogs presented peri-articular new bone formation in the studied articular process joints, three affecting C6–C7, four at C7–T1, and one at T1–T2. Spondylosis deformans was observed in a total of 57 dogs, mostly in grade 1 (45 dogs) and less frequently in grade 2 (11 dogs) and 3 (only one dog). The most affected intervertebral disc was C7–T1 (29 dogs, 24 distributed in grade 1 and five in grade 2), followed by C6–C7 (13 grade 1, 4 grade 2, and 1 grade 3) and T1–T2 (8 grade 1, 2 grade 2). Intervertebral disc herniation was only observed in a single dog, which was a Chihuahua with a grade 2 herniation located at the C6–C7 intervertebral disc. In the same dog, the affected intervertebral disc space also collapsed (Figure 7). Intervertebral disc mineralization was observed in a total of 85 dogs, with comparable distribution between different intervertebral discs (25 at C6–C7, 39 at C7–T1 and 21 at T1–T2).

Vertebral morphological abnormalities were only observed in two dogs. In one, there was enlargement of the C6 right transverse process without resulting in overt vertebral body malformation, and in the other dog, hypoplasia of the left cranial articular process of T1 was observed.

Organized information is provided in Supplementary Table S2.

### 3.4. Correlation of EPJA with Breed, Age, Sex, Body Weight, and Other CT Abnormalities

Most of the dogs (80%) with EPJA were purebreds, distributed in three Labrador Retrievers, two American Staffordshire Terrier, one Golden Retriever, one Bouvier des Flandres and one Chihuahua, with only one affected dog within the chondrodystrophic group. The remaining dogs (two, 20%) were crossbreeds. Except for one Labrador that had EPJA in two IVD (C7–T1 and T1–T2), all remaining dogs had only one endplate affected. The only breed correlations with EPJA were related to non-chondrodystrophic and purebred status (with no specific breed correlations).

The Chihuahua and the two crossbred dogs were in the small (<10 kg) breed group, while all other dogs were in the large (25–49 kg) breed group. No medium or giant dogs had EPJA. No correlation was observed between body weight and the presence of EPJA.

The only Type C EPJA was observed in an adolescent (1–2 years old) Labrador Retriever. All remaining dogs with EPJA were grouped in the adult or senior groups, with most of them (seven, 70%) placed in the senior group and two in the adult group. However, no significant correlation was observed when assessing age groups independently.

As for sex distribution, there was almost equal distribution between males and females: six males (60%) presented with a total of seven EPJA (63.6%), and four females (40%) presented with a total of four EPJA (36.4%), with no correlation between the presence of EPJA and sex (Table 1).

All dogs (100%) with EPJA showed the presence of other vertebral abnormalities in the same intervertebral disc space. All endplates with EPJA had sclerosis, and other visualized abnormalities were spondylosis deformans (three cases, 27.3%), intervertebral disc mineralization (three cases, 27.3%), osteoarthrosis (one case, 9.1%), and intervertebral disc herniation (one case, 9.1%). In four cases (36.4%), two concurrent abnormalities were present (sclerosis and sclerosis and spondylosis, sclerosis and IVD mineralization, and sclerosis and osteoarthrosis), and the single patient (Chihuahua) with intervertebral disc herniation also had concurrent endplate sclerosis and spondylosis deformans. The other two dogs with spondylosis deformans were a Labrador and a crossbred. The other dogs with IVD mineralization were an American Staffordshire and a Labrador Retriever, and the only patient with articular process joint osteoarthrosis was a Golden Retriever. No dogs showed vacuum phenomena or morphological abnormalities in the intervertebral disc spaces affected by EPJA. The only positively correlated (*p* < 0.05) factors with EPJA were the presence of at least one vertebral abnormality and endplate sclerosis. However, when independently assessing the other factors, no positive correlation was observed with the presence of presumed EPJF (Table 2).

## 4. Discussion

This paper describes the presence of EPJA as part of degenerative changes of the cervicothoracic spine, which could lead to the progression of degenerative changes and as an alternative method for intervertebral disc extrusion. In the current study, only 1.16% of endplates (3.2% of the dogs) showed EPJA on CT, with only one dog with concurrent disc herniation (C6–C7).

The main vertebral lesions affecting the endplate junction include osteochondrosis (including osteochondritis dissecans and osteochondral fragmentation), endplate junction failure, emerging spondylosis deformans, or spondyloarthritis. Similar to previous studies [4,13] that assessed the presence of presumed EPJF, the most commonly visualized EPJA was Type A, including the only dog with a confirmed intervertebral disc herniation.

Due to lesion characteristics and localization, osteochondral abnormalities such as osteochondrosis (OC) should be considered, especially in younger dogs. The distinction between osteochondrosis and other EPJA based on diagnostic imaging techniques is a potential challenge, also affected by the co-occurrence of other intervertebral or vertebral changes. Even if the nature of the different lesions differs, with OC presented as a developmental disorder and others presented as an acquired (possibly traumatic or degenerative) disorder, the characteristics of diagnostic imaging techniques are almost identical. The main difference relies on the time of presentation of the intervertebral disc and regional degeneration—in OC, the degenerative changes will appear secondary to osteochondrosis, while in the remaining, the intervertebral disc degeneration can act as the initial factor. However, since usually all findings are observed together, the distinction between original and secondary lesions remains elusive.

Based on publications in man [17,18], another differential diagnosis for Type A EPJA would be spondyloarthritis, which consists of chronic inflammatory disease that can be visualized as slight bone proliferation at the vertebral corner, including the endplate contour. However, this has not yet been described in dogs, and it can also look like mild or emerging spondylosis deformans, which is a more commonly described vertebral abnormality in dogs. Since all mentioned EPJA can present as irregular contours of the endplate, the distinction between both disorders can be challenging from an imaging point of view, and a definitive diagnosis would be linked to (histo)pathological analysis. Furthermore, both disorders could also be present at the same time, or one could lead to the progression of the other. Further studies could potentially aid in the differentiation of both processes.

Spondylosis deformans, a recognized [19] vertebral abnormality consisting of a chronic, degenerative, non-inflammatory disease characterized by osteophyte production at the ventral aspect of the vertebral endplates, can result in a complete bony ridge between vertebrae. In the current paper, when an abnormality was clearly identified as spondylosis, it was graded and classified as such, along with the other degenerative abnormalities of the spine. However, as already mentioned, in its initial stages spondylosis can resemble EPJA since it can occur as very mild contour irregularities.

Presumed EPJF has been described as failure of the attachment of the intervertebral disc annulus in the vertebral endplate, potentially leading to intervertebral disc herniation. Imaging characteristics have been described in human and veterinary medicine, and based on characteristics and location, it can look like osteochondrosis (OC) or spondylarthritis. In human medicine, no EPJF has been described in the cervical spine or cervicothoracic junction, with all studies focusing on the lumbar and lumbosacral spine. In those studies, all endplate failures were observed in the posterior aspect of the endplate, with no similar abnormalities observed in the anterior aspect. In our study, almost equal distribution was observed between dorsal and ventral locations. This could be explained by the different standing positions between the two species (biped versus quadruped) and subsequent force distribution along the intervertebral disc and endplate. This being said, in our population, only one dog had an EPJA with concurrent disc herniation, with the correlated site of herniation and EPJA lesion. For the dogs with lesions located at the ventral aspect of the endplate, this would not lead to herniation of disc material through the visualized defect due to the really low likelihood [20] of ventral herniation. However, this could indicate an overall weakness of the endplate-IVD union, maybe predisposing to future herniations [3,10,11].

Definitive diagnosis for the described types of EPJA should include histological examination, either after surgical excision or during post-mortem pathology. However, surgical therapy is not always available or performed, and (histo)pathological analysis may also not provide a definitive answer in some of the cases.

The fact that only one case was observed at T1–T2, with the remaining visible at the cranial part of the assessed spine, is likely related to the fact that the most flexible aspect of the cervicothoracic junction is located at C6–T1, while T1–T2 is already within the more static cranial thoracic spine.

Despite the inclusion of multiple dogs of breeds predisposed to CSM, no CT abnormalities compatible with CSM were observed in any dog. This is most likely related to the institutional preference to perform magnetic resonance imaging (MRI) in dogs suspected of CSM. Similar results were observed in dogs evaluated for herniated discs, with only one dog included in the study presenting a cervical disc herniation. This is also most likely related to the preference for MRI in dogs with neck pain and/or suspected herniated discs. In the current study, CT was chosen over MRI due to its inherently better quality in assessing bony structures, even though the trade-off is a decreased detail of soft tissue structures (such as the intervertebral disc). Due to the increased availability and performance of high-field MRI scans in veterinary medicine, future research focused on MRI changes or a combination of CT and MRI could be of value for the work-up and treatment of these dogs.

Since patients included in the study had CT performed for a variety of reasons, the fact that most of the affected dogs were non-chondrodystrophic could be related to a lower prevalence of chondrodystrophic patients in the included population. It could also be related to a lower predisposition of chondrodystrophic patients for EPJA in the cervicothoracic junction since those patients are more predisposed to cranial cervical abnormalities [5,6,7,9]. The low number of chondrodystrophic dogs in the current study might result in low statistical power and unclear relevance for this finding. Further research, including a higher number of chondrodystrophic dogs, is recommended to highlight potential differences.

The main reason for the high number of Labrador Retrievers included in the study is institutional workload. In the investigated veterinary hospital, multiple CT studies are performed for purchase screenings for helping dogs, which are young adult Labrador Retrievers (or Labrador crossbreeds) in a high percentage.

The main limitations of this study were the low number of dogs with EPJA and/or intervertebral disc herniation, the high proportion of young dogs presented for screening, and the retrospective nature of the study. The low number of intervertebral disc herniations is related to the already mentioned institutional preference for MRI in dogs with suspected spinal disease. The high percentage of dogs for screening is related to the high caseload of the institution for training and/or breeding programs. The retrospective nature of the study is related to the lack of (histo)pathology for any of the abnormal endplates, including the single case with an intervertebral disc herniation. Furthermore, obtaining endplate samples in dogs with no clinical complaints or disc herniation would be an excessively invasive diagnostic technique. However, differentiating mineralized fragments from osteochondrosis can be challenging, even in histology. In human medicine, post-contrast diffusion MRI has been described to identify the presence of endplate breaks; however, this is a time-consuming technique since the published studies require 120 min for adequate image assessment and have not been optimized in companion animals.

Another limitation of this study is the partial blinding of the CT reviewer. While CT scans were assessed blinded to signalment and clinical history, the same reviewer initially screened cases for inclusion, requiring access to patient records. This introduces potential selection and observer bias. Selection bias may arise if prior knowledge of clinical history influences which cases are included. Observer bias is possible if awareness of clinical signs subconsciously affects the interpretation of endplate alterations. Future studies could improve blinding by using an independent reviewer for case selection or fully anonymizing CT data before assessment.

## 5. Conclusions

In conclusion, cervicothoracic EPJA is only observed in a very low number of intervertebral disc spaces (1.16%) and patients (3.2%). It is always related to the presence of at least one other CT sign of intervertebral disc degeneration, with equal distribution between dorsal and ventral lesions. More research is needed to investigate possible clinical implications.

## Figures and Tables

**Table 1:** Population distribution of dogs with EPJA.

Breed	Labrador Retriever (3)
	American Staffordshire (2)
	Crossbred (2)
	Golden Retriever (1)
	Bouvier des Flandres (1)
	Chihuahua (1)
Sex	Male (4)
	Male neutered (2)
	Female (2)
	Female neutered (2)
Age	Senior (7)
	Adult (2)
	Adolescent (1)
Body Weight	Large (7)
	Medium (0)
	Small (3)

**Table 2:** Variables associated with the presence of EPJA.

Variable	Categories	*p*-Value
Age	Adolescent (1)	0.140
	Adult (2)	0.193
	Senior (7)	0.092
Body weight	Small (3)	0.096
	Medium (0)	0.210
	Large (7)	0.082
	Giant (0)	0.230
Breed	Purebred (8)	0.002
	Non-chondrodystrophic (9)	0.004
Endplate sclerosis	Present (11)	0.000
Other CT abnormalities	Present (8)	0.002

## Data Availability

Data is contained within the article.

## Supplementary Materials

The following supporting information can be downloaded at https://www.mdpi.com/article/10.3390/ani15081171/s1. Supplementary Table S1: complete list of breeds included in the study; Supplementary Table S2: Other CT abnormalities.

## Abbreviations

The following abbreviations are used in this manuscript:

EPJA	Endplate Junction Alteration
CT	Computed Tomography
AF	Annulus Fibrosus
AFF	Annulus Fibrosus Failure
NP	Nucleus Pulposus
MRI	Magnetic Resonance Imaging
IVD	Intervertebral disc
PACS	Picture Archiving and Communication System
ECVDI	European College of Veterinary Diagnostic Imaging
CSM	Cervical Spondylomyelopathy
EPJF	Endplate Junction Failure
OC	Osteochondrosis
