# Supplementary material for: Computed Tomography Prevalence of Cervicothoracic Endplate Junction Alterations in Dogs
Source: Animals (Basel). 2025 Apr 18;15(8):1171. doi: 10.3390/ani15081171 (PMC12023942; doi:10.3390/ani15081171)
Supplement: Supplementary file 1 [file animals-15-01171-s001.zip › animals-3557519-supplementary.pdf]

Supplementary Table S1. Complete list of breeds included in the study	
**BREED**	**NUMBER**
Crossbreed	90
Labrador Retriever	62
Golden Retriever	19
Belgian Shepherd	10
German Shepherd	9
American Staffordshire	8
Rottweiler	8
King Poodle	7
Labradoodle	6
Daschund	6
Rhodesian Ridgeback	5
Beagle	4
Bernese Mountain Dog	4
American Bulldog	3
Border Collie	3
Boxer	3
Chihuahua	3
Doberman	3
French Bulldog	3
Vizsla	3
Yorkshire Terrier	3
Australian Shepherd	2
Bullmastiff	2
German Wirehaired Pointer	2
Goldendoodle	2
Jack Russell	2
Leonberger	2
Swiss White Shepherd	2
American Cocker Spaniel	1
Australian Cattle Dog	1
Basset Hound	1
Bearded Collie	1
Beauceron	1
Bichon Frise	1
Boston Terrier	1
Bouvier des Flandes	1
Briard	1
Carin Terrier	1
Chow Chow	1
Dalmatian	1
Dutch Spaniel	1
English Springer Spaniel	1
Epagneul Bleu de Picardie	1
Fox Terrier	1
Giant Schnauzer	1
Greyhound	1
Irish Softcoated Terrier	1
Maltese	1
Mastino Napoletano	1
New Scotland Retriever	1
Newfoundlander	1
Pinscher	1
Pitbull	1
Poodle	1
Pug	1

Scottish Terrier	1
Shapendoes	1
Shetland Sheepdog	1
Shiba Inu	1
Shih Tzu	1
Shiloh Shepherd	1
Stabyhound	1
Staffordshire Bullterrier	1
Tibetan Terrier	1
Weimaraner	1
Welsh Springer Spaniel	1
Whippet	1

Supplementary Table S2. Other CT abnormalities

	C6-C7	C7-T1	T1-T2
**Endplate sclerosis**	No (302)	No (296)	No (309)
	Yes (13)	Yes (19)	Yes (6)
**Vacuum phenomenon**	No (311)	No (313)	No (314)
	Yes (4)	Yes (2)	Yes (1)
**Facet joint osteoarthrosis**	No (312)	No (311)	No (314)
	Yes (3)	Yes (4)	Yes (1)
**IVD mineralization**	No (302)	No (302)	No (302)
	Yes (13)	Yes (13)	Yes (13)
**IVD hernia**	Grade 0 (314)	Grade 0 (315)	Grade 0 (315)
	Grade 1 (0)	Grade 1 (0)	Grade 1 (0)
	Grade 2 (1)	Grade 2 (0)	Grade 2 (0)
	Grade 3 (0)	Grade 3 (0)	Grade 3 (0)
**Spondylosis deformans**	Grade 0 (297)	Grade 0 (286)	Grade 0 (305)
	Grade 1 (13)	Grade 1 (24)	Grade 1 (8)
	Grade 2 (4)	Grade 2 (5)	Grade 2 (2)
	Grade 3 (1)	Grade 3 (0)	Grade 3 (0)
